# Supplementary material for: The association of different presentations of maternal depression with children’s socio-emotional development: A systematic review
Source: PLOS Glob Public Health. 2023 Feb 27;3(2):e0001649. doi: 10.1371/journal.pgph.0001649 (PMC10021281; doi:10.1371/journal.pgph.0001649)
Supplement: S1 Text — (PDF) [file pgph.0001649.s002.pdf]

S2. Search results on February 07th, 2022

1. (depress* adj3 (mother* or maternal or postpartum or post-partum or postnatal or post-natal or puerperal or puerperium or perinatal or peri-natal or antenatal or ante-natal or antepartum or ante-partum or prenatal or pre-natal or prepartum or pre-partum)).mp. (54944)
2. ("trajector*" or "chronic*" or "recurrent" or "concurrent" or "repeated exposure").mp. (5382458)
3. 1 and 2 (5415)
4. ("longitudinal" or "longitudinal studies" or "follow-up" or "follow-up studies" or "follow up" or "follow up studies" or "followup" or "followup studies" or "prospective" or "prospective studies" or "cohort" or "cohort studies").mp. (7457249)
5. 3 and 4 (2524)
6. ("socioemotion*" or "socioemotion* develop*" or "socio-emotion* develop*" or internal* or external* or "socioemotion* function*" or "socio-emotion* function*" or "social emotional learning" or "emotion* develop*" or "psychosocial* develop*" or "psycho social* develop*" or "social adjust*" or "child* behavi*r" or "child* psycho*").mp. (2586181)
7. 5 and 6 (695)
8. ("child*" or "preschool" or "pre-school" or "school*").mp. (7467579)
9. 7 and 8 (664)
10. limit 9 to yr="1992 -Current" (659)
11. remove duplicates from 10 (352)
