# Supplementary material for: The association of different presentations of maternal depression with children’s socio-emotional development: A systematic review
Source: PLOS Glob Public Health. 2023 Feb 27;3(2):e0001649. doi: 10.1371/journal.pgph.0001649 (PMC10021281; doi:10.1371/journal.pgph.0001649)
Supplement: S2 Text — (PDF) [file pgph.0001649.s003.pdf]

S3. Data extraction pilot

A data extraction tool was created and piloted. Information was extracted on (i) study characteristics (title, name of the first author, year of publication, location, language, name of the cohort, number of follow-ups); (ii) sample characteristics (sample size, ages); (iii) assessments characteristics (timing of data collection, assessment tool used for mothers and offspring, cut-off used for each instrument, type of informant); and (iv) study results (MD presentation, type of methodology used to report MD presentation, reported significance values of the association between MD and children's internalising, externalising, and social-competence outcomes, effect sizes, adjusted covariates).
