# Supplementary material for: The association of different presentations of maternal depression with children’s socio-emotional development: A systematic review
Source: PLOS Glob Public Health. 2023 Feb 27;3(2):e0001649. doi: 10.1371/journal.pgph.0001649 (PMC10021281; doi:10.1371/journal.pgph.0001649)
Supplement: Attachment — Submitted filename: Response to Reviewers.docx [file pgph.0001649.s004.docx]

**PLOS Global Public Health: Reviewer comments and responses**

**Title**: The association of different presentations of maternal depression with children's socio-emotional development: A systematic review

**Manuscript number**: PGPH-D-22-00343

| **REVIEWERS RECOMMENDATIONS** | **AUTHORS RESPONSES** |
| --- | --- |
| **INTRODUCTION** | |
| **Reviewer 1:**  Line 33, why "thus" why is stress automatically a risk factor for offspring development? | We thank the reviewer for their comment. This sentence has been edited to: *“Exposure to additional perinatal challenges – such as maternal depression (MD) – may increase perinatal stress, potentially representing a risk factor for the development of suboptimal developmental outcomes in their offspring*” (Page 3). |
| **Reviewer 1:**  What is biological sensitization across the placental barrier, I find this a vague concept | Thank you for highlighting these issues. This sentence has been modified to: *“Depressive symptoms during pregnancy may affect the intrauterine environment and change immature biological systems in the foetus, potentially increasing the risk for future disease [6]”.* (Page 3). |
| **Reviewer 1:**  Please also briefly introduce the continuum concept of symptoms across severity. | Thank you for your suggestion. We have added the following to the introduction section: *“In addition to timing and chronicity of depressive symptoms, there is evidence that severity of MD is also associated with offspring difficulties [32, 40, 41]. Severity of depressive symptoms includes a continuum of problems with different levels of symptoms, including lower, moderate, and severe levels over time.”* (Page 5).  Additionally, in the operational definitions section, severity is defined as: *“Severity: a continuous gradient reflecting variation of depressive symptoms, including low, moderate, and severe levels. Fluctuation of symptoms over time can be reflected by increasing, decreasing, or constant symptoms.”* (Pages 8-9). |
| **Reviewer 1:**  Is there really good evidence that MD is an independent risk factor for ext and int, have longitudinal studies using repeated measures and cross-lagged panel designs or so been performed? | Yes, there are longitudinal studies with repeated measures reporting MD as independent risk factor. In line with the reviewer’s suggestion, the following has now been included: *“Additionally, longitudinal studies using repeated measures reported that maternal depressive symptoms during the antenatal and postnatal period were associated with elevated rates of externalising [8, 12, 18, 19], internalising [18, 20], peer relationship problems and lower prosocial behaviours [21], along with difficulties reaching overall developmental milestones in childhood [22]”* (Pages 3-4). |
| **Reviewer 2:**  Important, large-scale cohort studies from the ALSPAC cohort, Generation R cohort, PREDO cohort, MOBA cohort and Dream Big Consortium need to be added as study references and addressed in the systematic review before the conclusions of the review are justified. For example, the ALSPAC studies, for example by O'Donnell et al. (2014) doi:10.1017/S0954579414000029 have addressed the children at multiple ages and include method-of-choice studies on the consequences of maternal perinatal depression on child internalizing and externalizing problems. The PREDO study by Lahti et al. (2017) assessed children at an average age of 3.5 years and should be included in the review. Wolford and colleagues addressed ADHD symptoms in children over 3 years of age. A recent publication from the Dream Big Consortium by Szekely et al. (2021) https://doi.org/10.1016/j.jaac.2020.02.017 have elaborately summarized study findings on the addressed topic from ALSPAC, Generation R and MAVAN cohorts. Gjerde and colleagues have in multiple publications addressed the consequences of maternal depression on child behaviour in the MOBA cohort.  Please also carefully go through the review by Van Den Bergh et al. (2020): Prenatal developmental origins of behavior and mental health: The influence of maternal stress in pregnancy and Robinson et al. (2019: https://doi.org/10.1038/s41390-018-0173-y and Madigan et al. 2018: doi: 10.1016/j.jaac.2018.06.012 for more studies on the addressed study topic.  Before all the necessary studies are included, the review is not systematic but selective. In its current format, the reviewer is uncertain what the key addition of the current review to the study literature is, given there are multiple reviews on this study topic, some of which are more comprehensive than the current one. | We thank the reviewer for raising these issues. We acknowledge that the maternal depression topic is similar to previous reviews. However, this study adds to previous work by considering the complexity of different presentations of maternal depressive symptoms from the perinatal period onwards, challenging the classic conceptualisation of maternal depression as a homogeneous construct. Moreover, the results of this review also highlighted common methodological problems and provided future directions for research on the topic.  We carefully revised the recommended studies from the reviewer and included the Wolford et al. (2017) and Gjerde et al. (2017) studies. These studies were unavailable during the screening process because they did not appear in the keyword selection, and we apologise for this oversight. Therefore, they were added as ‘Additional articles identified through other sources’ in the PRISMA flow diagram.  Conversely, considering our inclusion and exclusion criteria, we did not include the other suggested studies in the review. More precisely:   - The O’Donnell et al. (2014) and Szekely et al. (2021) studies measured maternal depression only in the perinatal period (antenatal and postnatal stages), without including another assessment after the postnatal period. - Lahti et al. (2017) included children from 1.9 to 5.9 years old, without reporting individual results per year of age. Therefore, the study included a broader age range than the systematic review inclusion criteria.   We also looked at the included studies in the systematic reviews and meta-analysis from van Den Bergh et al. (2020), Madigan et al. (2018) and Robinson et al. (2019). However, the three of them only measured the antenatal period. Therefore, their studies were excluded from this review.  Nevertheless, we acknowledge the importance of those excluded studies for our findings. Consequently, they were added as references in the introduction and discussion sections. |
| **METHODS** | |
| **Reviewer 2:**  If exclusion has been done based on public availability of the studies, the authors could have very well at least included key results from studies where this data was not available due to data sensitivity issues. | We are not clear what the reviewer is asking. There are no exclusion criteria related to public availability of studies, also this type of situation was not stated in the PRISMA flow diagram. |
| **Reviewer 2:**  The rationale for excluding children with age below 3 years is not very thoroughly justified, as available instruments such as CBCL do assess child externalizing and internalizing problems from 1.5 years of age onwards and these early symptoms do have predictive value for later mental health also. It also remain unclear whether average age of the child is the key criterion for exclusion and what was done in studies where the whole age range was not reported | We appreciate the reviewer’s concern. However, the rationale for selecting children’s outcomes from three years onwards was to target children from the preschool period. Internalising problems are more challenging to detect in infants or toddlers because of their less developed verbal skills, and social competence can be difficult to assess when children have fewer opportunities to engage with peers. Therefore, even if measurements such as the CBCL have information on previous developmental ages, we preferred to select children from the preschool period. Moreover, adjusting the age lower bound would imply changing the eligibility criteria, which would be of concern given that the protocol has already been published.  However, we acknowledge that some important studies were excluded for this reason. Therefore, it was included as a study limitation: *“First, our inclusion criteria included children from three years onwards only; several otherwise eligible studies on the topic were excluded based on this criterion [e.g., 12, 88]. However, the rationale for targeting children from the preschool period onwards reflected the challenges in detecting internalising problems in infants or toddlers, arising due to less developed verbal skills, and social competence can be difficult to assess when children have fewer opportunities to engage with peers [89]. Therefore, even if included studies reported measurement data on previous ages, we did not report these.”* (Page 37).  Children’s age in the assessment timepoint is one of the study inclusion criteria *(“assessing the associations with children’s internalising, externalising and social competence behaviours (e.g., social skills, peer relationship, prosocial behaviour) at least once from three to twelve years old”*), not the average age. Because children’s age was an inclusion criterion, studies which did not report this information were excluded in the selection stage. |
| **Reviewer 2:**  Additionally, the definition of social competencies should be pointed out more clearly-which studies are included here. | Thank you for your comment. In line with the reviewer’s suggestion, the eligibility criteria section has been edited to: *“assessing the associations with children’s internalising, externalising and social competence behaviours (e.g., social skills, peer relationship, prosocial behaviour) at least once from three to twelve years old”).* (Page 6). |
| **Reviewer 2:**  A Newcastle Ottawa Scale assessment by two independent reviewers should be conducted on the validity of the included studies, but only after all necessary studies are included in the review. | We thank the reviewer's recommendation. However, we have already conducted a quality assessment using the Agency for Healthcare Research and Quality (AHRQ) checklist, which has also been used for previous systematic reviews and meta-analyses (e.g., Raouna et al., 2018; Sweeney & MacBeth, 2016). Moreover, most of the items suggested in the Newcastle Ottawa Scale assessment were also assessed with the AHRQ (e.g., selection of the cohort, control groups, control for additional factors, outcome assessment blind to the exposure). Finally, the AHRQ was already published as the quality assessment tool in the systematic review protocol. We have discussed quality extensively in the discussion and tightened up language here in relation to the reviewers concern. |
| **RESULTS** |  |
| **Reviewer 1:**  It seems the authors do not review the quality of the study base (general population) or recruitment strategies, this is important as associations in convenience samples may be biased or not generalizable. | We appreciate the reviewer’s concern. We have added the following to the results section: *“Seven studies reported more than 30% attrition, and all of them included steps to minimise bias except for three [58, 62, 65]. Some studies reported attrition patterns suggesting more disadvantaged families (e.g., younger, less educated, with less financial resources mothers) had greater drop-out from follow-up than less diasvantaged families [30-31, 35-36, 53, 54, 56, 60, 63, 66]. The majority of studies did not report control groups (i.e., groups of women without depressive symptoms).”* (Page 28).  And in the discussion section: *“In addition, ten studies reported a pattern of attrition where more disadvantaged families were underrepresented. Thus, results should be interpreted carefully when generalising a broader population.”* (Page 37). |
| **Reviewer 1:**  Page 27, line 319, I fail to understand the concept of control groups in continuous symptom score studies | Thank you for highlighting these issues. This sentence has been modified to: *“The majority of studies did not report control groups (i.e., groups of women without depressive symptoms).”* (Page 28). |
| **Reviewer 2:**  In assessment of study quality and discrepant study findings, careful consideration of the statistical power due to differences in sample size should be taken into account. The studies using teacher reports have generally had much smaller sample sizes. | Thank you for your helpful suggestions. We have added the following to the discussion section: *“Discrepancies may also reflect methodological constraints since most of the studies using teacher reports included smaller sample sizes (e.g., [30, 59, 62]) than studies using maternal information, impacting upon statistical power to detect significant associations.”* (Page 32).  It’s worth noticing that reviewers 1 and 2 have different interpretations regarding the results of studies using teachers’ assessments (i.e., share method variance vs statistical power). Therefore, we tried to acknowledge both potential explanations in the manuscript. |
| **DISCUSSION** | |
| **Reviewer 1:**  Page 31, line 386, I fail to understand the conclusion about the timing of MD in relation to externalizing problems, please clarify | Thank you for your comment. In line with the reviewer’s suggestion, this section has been edited to:  *“In a previous review, Grace, Evindar, and Stewart [83] identified inconsistent results in relation to the association between postnatal depression and externalising problems, suggesting the timing of depression in the postnatal period was insufficient to account for children’s externalising outcomes [37, 83].”* (Page 34). |
| **Reviewer 1:**  My main concern is the very timid discussion of shared or common method variance bias. In other words, many researchers now argue that we cannot use maternal information on depression and the outcome, as relying on the same rater will systematically and substantially bias=inflate associations. Indeed, different informant provide different views on the outcome (setting or relation) but cannot be compared or contrasted, if one of the informants also reports on the exposure. A very nice study on shared method variance bias on prenatal depression is from Ringoot et al. (admittedly the reviewer's own group): Ringoot, Ank P., Henning Tiemeier, Vincent WV Jaddoe, Pety So, Albert Hofman, Frank C. Verhulst, and Pauline W. Jansen. "Parental depression and child well-being: young children's self-reports helped addressing biases in parent reports." Journal of clinical epidemiology 68, no. 8 (2015): 928-938.  This study suggests that maternal outcome reports in this situation inflate the association by up to 50%. Thus, the discussion on page 30 and 31 must be adapted, expanded; this problem needs much more attention and the authors must be much more critical. Shared method variance bias is a major flaw in the field and several top journals, such as AJP, will not accept child studies with maternal exposure and outcome report, if the variables have a degree of subjectivity. see also: Madsen, Kathrine Bang, Charlotte Ulrikka Rask, Jørn Olsen, Janni Niclasen, and Carsten Obel. "Depression-related distortions in maternal reports of child behaviour problems." European Child & Adolescent Psychiatry 29, no. 3 (2020): 275-285.  In this context the negative studies using teacher reports should receive more, much more attention. | Thank you for your helpful suggestions. We have added the following to the discussion section: *“Several explanations may underpin this discrepancy between informants. First, maternal negative cognitions due to depressive symptoms may affect appraisals of one’s children’s behaviours and potentially over-report child problems [78]. As mothers are source of information on both the exposure (i.e., maternal depression) and the outcome (i.e., child problems), associations may be inflated due to shared method variance [79]. Specifically, in studies using the same informant for the exposure and outcome, associations between maternal depression and childhood problems may be explained by the characteristics of the informant (e.g., negative cognitions) rather than the depression exposure itself. For example, a study by Ringoot and colleagues [80] reported similar associations between maternal depression and childhood externalising and internalising problems using different informants, although the associations registered by parents with depressive symptoms were described as inflated. However, informant discrepancies may also reflect the impact of the school environment on children’s behaviours [81] and teachers’ preconceptions regarding children’s behaviours, further linked to broader social determinants of health [82]. Discrepancies may also reflect methodological constraints since most of the studies using teacher reports included smaller sample sizes (e.g., [30, 59, 62]) than studies using maternal information, impacting upon statistical power to detect significant associations.” (Pages 32-33).*  *“Thus, general conclusions about MD and its associations with children’s outcomes may be biased potentially due to shared method variance if they only consider maternal reports and do not incorporate other informants (such as teacher reports) and they should not be seen as evidence that MD causes poor children socio-emotional outcomes.”* (Page 36).  Limitations: “*Finally, most of the included studies relied on maternal reports of both maternal depression and children’s socio-emotional problems. Accordingly, due to potential overestimation of associations attributable to shared method variance, the findings should be interpreted with caution”* (Page 38).  Implications for research and practice: *“In relation to children’s assessment, we emphasise the importance of utilising different types of informants (e.g., teachers, children’s own perspectives, external examinators) since the choice of the informant may impact the results estimates”* (Page 37).  Moreover, as mentioned in a comment for reviewer 2, reviewers have different interpretations regarding the results of studies using teachers’ assessments (i.e., share method variance vs statistical power). Therefore, we tried to acknowledge both potential explanations in the manuscript. |
| **Reviewer 1:**   The authors argue that the association of maternal depression with externalizing disorders is stronger. In the absence of meta-analytical metrics and given the clearly overlapping effect estimates this interpretation is problematic. In contrast, I think we can see this review as providing evidence that there is a lack of specificity in outcomes. I argue this is typical for research on maternal depression and if the authors fail to agree, I would at least like to see a more critical and nuanced discussion. | We thank the reviewer for their comment. This section has been edited to: *“For internalising outcomes, effect sizes were mostly medium size, whereas for externalising and social competence outcomes, reported effect sizes were large. However, in the absence of meta-analysis, precise estimates of the effect of depression on outcomes is still lacking. On the other hand, our findings also provide evidence for a lack of specificity in children’s socio-emotional outcomes after maternal depression exposure. This may reflect the effect of one underlying psychopathology construct [75] or dependency across socio-emotional problems in childhood [76]. Future research should further explore predictors of overlapping outcomes.”* (Page 32). |
| **Reviewer 1:**  For clinical research the question to what extent the associations reflect genetic transmission of risk is important, this should be studied with modern genetic techniques and using paternal genetics/trio design. This deserves a paragraph in the discussion, many studies simply conclude there is evidence for both environmental and genetic transmission, this may not be true e.g. antenatally. | Thank you for your helpful suggestions. We have added the following to the discussion section: *“These associations may reflect both environmental (e.g., maternal sensitivity) and genetic (e.g., genetic confounding) transmission of risk. For example, Gjerde and colleagues [67] reported that associations between maternal depressive symptoms and children’s behavioural and emotional outcomes were explained through both shared genetic liability and direct environmental exposure, although with different relative importance throughout childhood. Future studies using modern genetic techniques such as trio designs may help to clarify the genetic contribution of maternal depressive symptoms at different stages in childhood.”* (Pages 35-36). |
| **Reviewer 1:**  Please discuss why latent classes and other grouping of symptoms make sense and a continuous analysis need not be preferred, discuss in the context of patterns of decrease and increase. | Thank you for your comment. In line with the reviewer’s suggestion, this section has been edited to: *“Further research on MD should continue assessing different presentations through a variable-centred or person-centred approach, allowing group allocation according to patterns within the population. These methods will help to better describe the course of maternal depressive symptoms, identifying different progressions that may not be identifiable using a priori categorisations. Studies using their own criteria or assignment rules to create MD groups have several limitations like assuming a priori the existence of different groups, missing the nuances of change over time (e.g., patterns of decreasing and increasing symptoms may be overlooked), and potentially confounding chronicity and severity. Therefore, looking at longitudinal change patterns instead of assumed categories may help to further disentangle symptom features such as chronicity, severity, and timing.”* (Page 38). |
| **Reviewer 1:**  The fluctuations of symptoms/transient groups should receive more attention, in particular the decrease in symptoms. | In line with the reviewer’s suggestion, the following has now been included: *“In addition, the majority of studies also identified transient groups such as increasing and decreasing symptoms, moderate or subclinical symptoms, and episodic depressive symptoms at some periods only. These groups are consistent with findings supporting a recovery group of mothers within the perinatal period [26], and a group at risk for future depressive symptoms, reporting symptoms up to three years after birth [26, 73]. Transient groups may reflect the emergence of further challenges beyond the perinatal period. For example, increasing symptoms groups may reflect the impact of children’s behavioural or emotional problems on maternal depressive status, with studies reporting bidirectional associations between maternal depressive symptoms and children’s behavioural problems [74]. In contrast to increasing groups, it was noted that women with decreasing symptoms tend to have lower depression levels by the time their children start school [30, 56], perhaps indicative of the social support role of school. Future research could unpack the differential impact of social determinants such as school setting on depressive symptoms across motherhood.”* (Pages 30-31). |
| **Reviewer 1:**  Was there any relation between quality of studies and effect size? | Thank you for your comment. In line with the reviewer’s suggestion, we have added the following to the discussion section: *“Finally, there was no clear relation between the quality of studies and effect sizes. The three studies with higher quality percentages reported large, medium, and small effect sizes, including a study using teachers’ reports, whilst the study with the lowest quality percentage also reported large effect sizes for internalising, externalising, and chronicity of symptoms.”* (Page 37). |
| **Reviewer 1:**  The authors conclude (p.31) that the association of antenatal symptoms with child outcomes is not consistent. That is true, but why not interpret this as evidence that the impact is less strong. This can also be a lead to the mechanisms of transmission. This is not black and white issue, but the authors should reconsider their interpretation. | We appreciate the reviewer’s concern. This section has been edited to: *“These results were mostly inconsistent with the argument that antenatal MD represents an independent risk factor for internalising and externalising outcomes [12, 14, 22]. This finding may suggest that the impact of antenatal depressive symptoms is weaker than the postnatal effect on children’s outcomes, indicating that pregnancy-specific effects are insufficient to explain childhood internalising, externalising and social-competence problems. Thus, other mechanisms of transmission may help to explain the MD and children’s socio-emotional problems associations, such as genetic transmission, exposure to the mother’s behaviours, and contextual stressors. However, there was selective attrition across follow-up in these studies – with women with depressive symptoms during pregnancy more likely to drop out from studies, impacting upon potential study power and underestimation of antenatal MD with its associated outcomes. Further, as only five studies report antenatal MD results and selective attrition across follow-ups, there is at present, insufficient evidence to evaluate the associations of antenatal MD on children’s internalising, externalising and social competence outcomes.”* (Page 34). |
| **Reviewer 1:**  Studies of concurrent depression rated by the mother should be omitted from the present study, they are not informative. | We thank the reviewer for their comment. The review’s aims included an examination of the associations between different presentations of maternal depression from the perinatal period with later stages of motherhood and children’s outcomes, along with the impact of the timing, chronicity, and severity of maternal depressive symptoms on children’s outcomes. Whilst studies were longitudinal in nature given the repeated measures required in order to fully address the second question, we do not see the benefit in removing information around concurrent associations as this provides a more complete account of the reviewed study findings. For example, results indicate large and medium effect sizes of current depression on children’s outcomes, and we therefore reflected that it is important to include these results considering clinical implications.  Regarding the point of potential bias in concurrent depression rated by the mother, degrees of bias are present with all self-report measures, but it is often what we must rely on within the social sciences and therefore we have acknowledged this potential limitation. |
| **Reviewer 1:**  Power calculations in observational studies are highly debated, I disagree they should be routinely reported, power can be deducted from the CI. | We appreciate the reviewer’s concern. We discussed power calculation as a function of the quality assessment tool utilised (Agency for Healthcare Research and Quality; AHRQ). However, we added the following to acknowledge this limitation: *“The main methodological issue across studies was the reporting and justification of sample size, where no studies reported a power analysis. However, the utility of power calculations in observational studies has been questioned [68], and power can be inferred from other sources as confidence intervals.”* (Page 27). |
| **Reviewer 1:**  How could studies disentangle severity and chronicity? Please discuss. | We thank the reviewer for their comment. We added the following in the discussion section: *“Further research on MD should continue assessing different presentations through a variable-centred or person-centred approach, allowing group allocation according to patterns within the population. These methods will help to better describe the course of maternal depressive symptoms, identifying different progressions that may not be identifiable using a priori categorisations. Studies using their own criteria or assignment rules to create MD groups have several limitations like assuming a priori the existence of different groups, missing the nuances of change over time (e.g., patterns of decreasing and increasing symptoms may be overlooked), and potentially confounding chronicity and severity. Therefore, looking at longitudinal change patterns instead of assumed categories may help to further disentangle symptom features such as chronicity, severity, and timing.”* (Page 38). |
| **Reviewer 1:**  Page 33, limitations: Inconsistency and heterogeneity of results is no limitation but a legitimate result. You did could work to show this. | We appreciate the reviewer’s concern. In line with the reviewer’s suggestion, this section has been changed and added in the second paragraph of the discussion: *“All studies described different typologies of MD, identifying between two and seven distinct MD groups. This confirms existing evidence identifying different types of MD presentations at different periods [5, 40, 70-72]. However, methodological inconsistencies between studies, particularly variation in the approach to assessment of MD groups may have produced substantial differences in the reported MD groups, and consequently hampers comparisons between studies. That said, identification of both a no/low depressive symptom group and a high chronic depressive symptom group was a common feature of the majority of identified studies.”* (Page 31). |
| **Reviewer 1:**  If paternal depression is suggested as future challenge to be studied, please mention the challenge of assortative mating, the influence of mothers on fathers (and vice versa); their effects are certainly not independent | Thank you for your comment. In line with the reviewer’s suggestion, this section has been edited to: *“Future research could also incorporate paternal depression different presentations over time, given their contribution to offspring internalising and externalising outcomes [90], including the potential interaction between mothers’ and fathers’ depressive symptomatology (e.g., the influence of the mother/father symptoms on the other partner’s wellbeing) or other challenges such as assortative mating [91].”* (Pages 38-39). |
| **OTHERS** | |
| **Reviewer 1:**  The term heterogeneity is used in reviews to describe studies that cannot easily be meta-analyzed and the term is used in studies of symptom heterogeneity, for example if different symptom patterns exist. Both is not addressed; thus, I recommend to avoid the term. | Thank you for highlighting these issues. The term has been modified across the manuscript. For example: *“*substantial variability in presentations of MD from the perinatal period onwards*”* (Page 31). |
| **Reviewer 1:**  Give LMIC in full in abstract | Thank you for your suggestion. This sentence has been modified to: *“Further research in low- and middle-income countries is warranted.”* (Page 2). |
| **Reviewer 1:**  Page 3 line 47, typo: have not 'has' | Thank you for highlighting this typo. This sentence has been modified to *“More precisely, both antenatal MD and postnatal MD have been associated with increased risk of adverse developmental and psychological outcomes in children [6, 11, 12]”* (Page 3). |
