# Supplementary material for: The association of different presentations of maternal depression with children’s socio-emotional development: A systematic review
Source: PLOS Glob Public Health. 2023 Feb 27;3(2):e0001649. doi: 10.1371/journal.pgph.0001649 (PMC10021281; doi:10.1371/journal.pgph.0001649)
Supplement: Attachment — Submitted filename: Response to Reviewers.docx [file pgph.0001649.s005.docx]

**PLOS Global Public Health: Reviewer comments and responses**

**Title**: The association of different presentations of maternal depression with children's socio-emotional development: A systematic review

**Manuscript number**: PGPH-D-22-00343

| **REVIEWERS RECOMMENDATIONS** | **AUTHORS RESPONSES** |
| --- | --- |
| **INTRODUCTION** | |
| **Reviewer 1:**   1. Line 52 and point 4 of my comments addressed: in the Introduction they speak of "independent" associations with internalizing and externalizing problems. The issue of independent association is not really discussed, how do the cited studies show that internalizing and not externalizing (or vice versa) symptoms drive the association? Or do they simply mean independent of confounding, that had not need to be mentioned? The first is more challenging and interesting and poorly addressed in the manuscript. Are we measuring the association with overlapping symptom presentations or independent associations? | We thank the reviewer for their comment. We referred to ‘externalising and/or internalising independent associations’ as independent from other confounding factors, such as repeated maternal depressive episodes in later developmental stages. Thus, in line with your concerns, the term independent has been removed from the introduction, and this section has been edited to: “For internalising symptoms, there is greater evidence for an effect of postnatal MD, which remains stable after accounting for repeated maternal depressive episodes in later developmental stages and environmental risks factors [14, 38]. Additionally, more recent longitudinal studies have demonstrated that antenatal and postnatal MD may constitute different risk factors for offspring externalising and internalising outcomes [12, 14, 18, 22, 39]. Therefore, MD may also lead to different pathways for transmission of risk depending on the specific period of onset of depression, highlighting the importance of the timing of symptoms when assessing children’s outcomes.” (Page 5).  However, we acknowledge the importance of highlighting that cited studies are not examining overlapping or independent associations with externalising and internalising outcomes. Therefore, the following point has been edited in the discussion section: *“On the other hand, our findings also provide evidence for a lack of specificity in children’s socio-emotional outcomes after maternal depression exposure. This may reflect the effect of one underlying psychopathology construct [75] or dependency across socio-emotional problems in childhood [76]. However, because cited studies did not examine overlapping presentations, questions about potential independent associations with externalising or internalising outcomes remain. Thus, future research should further explore if these associations represent overlapping symptom presentations or independent associations.”* (Page 32) |
| **RESULTS** | |
| 2. Page 27, line 319, I fail to understand the concept of control groups in continuous symptom score studies Thank you for highlighting these issues. Answer: This sentence has been modified to: “The majority of studies did not report control groups (i.e., groups of women without depressive symptoms).” (Page 28).   I do not agree with this answer, in an analysis of continuous symptoms not only the persons with no symptoms can be viewed as "control" group, this concept does not exist and the expectation that studies report the number of persons who did not endorse any symptoms is inappropriate. | We thank the reviewer for raising these issues. We acknowledge that when analysing different presentations and continuous symptoms there may be different control groups, not necessarily the mothers with no symptoms. Consequently, in line with the reviewer’s concerns, this section has been removed *(“The majority of studies did not report control groups (i.e., groups of women without depressive symptoms”*). |
| 5. However, the utility of power calculations in observational studies has been questioned [68], and power can be inferred from other sources as confidence intervals.”   Should be "such as" | Thank you for highlighting this issue. This sentence has been modified to: *“However, the utility of power calculations in observational studies has been questioned [68], and power can be inferred from other sources such as confidence intervals.”* (Page 27) |
| **DISCUSSION** | |
| 3. What does it mean: "suggesting the timing of depression in the postnatal period was insufficient to account for children’s externalising outcomes"?   Insufficient exposure duration? I fail to understand. | We thank the reviewer for their comment. This section refers to a previous review conducted by Grace, Evindar, and Stewart (2003), in which they concluded that the effects of postpartum depression on children’s externalising outcomes are likely to be explained due to chronic depression exposure rather than postnatal depression per se. To improve clarity, this section has been edited to: *“In a previous review, Grace, Evindar, and Stewart [83] identified inconsistent results in relation to the association between postnatal depression and externalising problems, suggesting the timing of depression in the postnatal period was not enough to account for children’s externalising outcomes, and that other factors such as chronic exposure may better explain these associations [37, 83].”* (Page 34).  Grace SL, Evindar A, Stewart DE. The effect of postpartum depression on child cognitive development and behavior: A review and critical analysis of the literature. Arch Womens Ment Health. 2003; 6:263-274. <https://doi.org/10.1007/s00737-003-0024-6> |
| 4. However, in the absence of meta-analysis, precise estimates of the effect of depression on outcomes is still lacking.   "are lacking" | We thank the reviewer for their comment. This section has been edited to: *“However, in the absence of meta-analysis, precise estimates of the effect of depression on outcomes are still lacking”* (Page 32) |
| 6. Thus, other mechanisms of transmission may help to explain the MD and children’s socio-emotional problems associations, such as genetic transmission, exposure to the mother’s behaviours, and contextual stressors.   "must also at least partly explain" | Thank you for your comment. In line with the reviewer’s suggestion, this sentence has been edited to: *“Thus, other mechanisms of transmission must also at least partly explain the MD and children’s socio-emotional problems associations, such as genetic transmission, exposure to the mother’s behaviours, and contextual stressors.”* (Page 34) |
| 7. Not sure I like the terms "increasing groups", be more descriptive, this is a bad label, term it symptoms group such as "transient symptom group" | Thank you for your suggestion. In line with the reviewer’s suggestion, the discussion section has been edited to: *“Transient symptom groups may reflect the emergence of further challenges beyond the perinatal period. For example, transient increasing symptoms groups may reflect the impact of children’s behavioural or emotional problems on maternal depressive status, with studies reporting bidirectional associations between maternal depressive symptoms and children’s behavioural problems [74]. In contrast, it was noted that women with transient decreasing symptoms tend to have lower depression levels by the time their children start school [30, 56], perhaps indicative of the social support role of school.”* (Pages 31-32) |
